# Quorum Sensing Inhibitors to Quench *P. aeruginosa* Pathogenicity

**DOI:** 10.3390/ph14121262

**Published:** 2021-12-05

**Authors:** Marine Duplantier, Elodie Lohou, Pascal Sonnet

**Affiliations:** AGIR, UR4294, UFR of Pharmacy, Jules Verne University of Picardie, 80037 Amiens, France; marine.duplantier@etud.u-picardie.fr (M.D.); elodie.lohou@u-picardie.fr (E.L.)

**Keywords:** anti-bioresistance, *Pseudomonas aeruginosa*, biofilms, virulence factors, anti-virulence strategy, bacterial communication systems, quorum sensing, quorum sensing inhibitors

## Abstract

The emergence and the dissemination of multidrug-resistant bacteria constitute a major public health issue. Among incriminated Gram-negative bacteria, *Pseudomonas aeruginosa* has been designated by the WHO as a critical priority threat. During the infection process, this pathogen secretes various virulence factors in order to adhere and colonize host tissues. Furthermore, *P. aeruginosa* has the capacity to establish biofilms that reinforce its virulence and intrinsic drug resistance. The regulation of biofilm and virulence factor production of this micro-organism is controlled by a specific bacterial communication system named Quorum Sensing (QS). The development of anti-virulence agents targeting QS that could attenuate *P. aeruginosa* pathogenicity without affecting its growth seems to be a promising new therapeutic strategy. This could prevent the selective pressure put on bacteria by the conventional antibiotics that cause their death and promote resistant strain survival. This review describes the QS-controlled pathogenicity of *P. aeruginosa* and its different specific QS molecular pathways, as well as the recent advances in the development of innovative QS-quenching anti-virulence agents to fight anti-bioresistance.

## 1. Introduction

The emergence and the dissemination of multidrug-resistant (MDR) bacteria constitute a major public health issue. Currently, microbial resistance infections are responsible for 700,000 death per year in the world, as specified in the review on antimicrobial resistance [1]. According to the annual European Centre for Disease Prevention and Control’s (ECDC) epidemiological report, at least 670,000 infections were due to antibacterial resistant strains in the European Union or European Economic Area (EU/EEA) countries in 2019 [2]. These infections are responsible for nearly 33,000 deaths and a cost for healthcare systems of around 1,1 billion euros. Among incriminated Gram-negative bacteria, *Pseudomonas aeruginosa* has been designated by the WHO as a critical priority threat. This pathogen is responsible for various nosocomial infections, usually lethal for patients suffering from cystic fibrosis. Its important genetic flexibility explains its prodigious phenotypical adaptability and its rapid acquisition of numerous antibiotic (ATB) resistance mechanisms [3]. This includes the production of enzymes able to degrade ATBs, such as β-lactamases and especially carbapenemases [4], a membrane permeability default due to porin deficiency, the implementation of efflux systems [5], and the modification of pharmacological targets [6]. Furthermore, *P. aeruginosa* has the capacity to establish biofilms that reinforce its virulence and intrinsic drug resistance. In order to regulate biofilm development, this pathogen uses a specific bacterial communication system named Quorum Sensing (QS). This sophisticated network of intra- and inter-species interactions relies on the secretion and perception of small signalling molecules called autoinducers (AIs) [7]. The intracellular concentration of AIs is modulated according to bacterial population density and external stimuli. The accumulation of AIs above a threshold concentration coordinates the expression of QS-associated genes via the activation of specific transcription factors. It induces the biosynthesis of essential proteins for microbial adaptation to environmental changes involving those implicated in the virulence pathways [8].

The threat of a post-ATBs era must encourage us to reinvent the anti-biotherapy. The development of anti-virulence agents (AVAs) that could attenuate pathogenicity of bacteria without affecting their growth, seems to be a promising new therapeutic strategy [9]. Indeed, the selective pressure put on sensitive bacteria by conventional antimicrobial molecules, causing their death, promotes resistant strain survival. Non-bactericidal AVAs could increase pathogen sensibility to the host immune system response in monotherapy. In combination therapy, they could restore the efficiency of current ATBs by inhibiting the formation of the hermetic barrier provided by biofilms. Of the most interesting approaches to quench virulence factor production, one approach is to target bacterial communication systems. This review describes the QS-controlled pathogenicity of *P. aeruginosa* and its different specific QS molecular pathways. Finally, we will highlight recent advances in the development of innovative QS-quenching AVAs to fight the anti-bioresistance.

## 2. QS-Controlled Pathogenicity of *P. aeruginosa*

**Figure 1:**
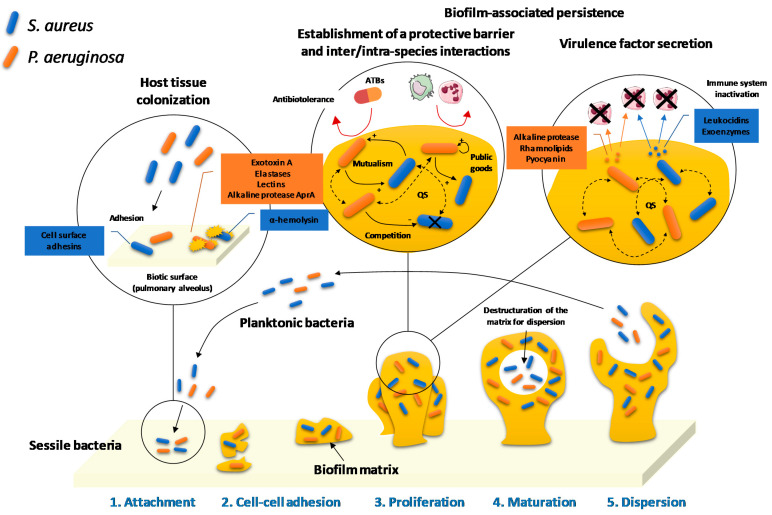
Host tissue colonization, biofilm development, invasion and persistence of *P. aeruginosa* during polymicrobial respiratory infections.

The infectious agent virulence is correlated to its pathogenic potential and depends on its ability to adhere and colonize host tissues, to escape the immune system response and to secrete toxins. *P. aeruginosa* has first at its disposal several physiological elements that constitute its primary arsenal. Indeed, its cell wall is surrounded by a weakly permeable outer membrane which constitutes of lipopolysaccharide (LPS) endotoxins and selective porins. This is associated with a flagella and *pili,* ensuring its adhesion to the substratum and its mobility; but also, different sophisticated secretion systems are implicated in the production of virulence factors. In addition to its capacity to establish biofilms, *P. aeruginosa* produces, under the control of QS, various proteolytic enzymes (elastases, alkaline proteases and type IV proteases), exotoxins (exotoxin A, exoenzymes and pyocyanin), siderophores (pyoverdines and pyochelin), rhamnolipids, hydrogen cyanide and lectins implicated in its pathogenicity. Furthermore, host tissue colonization is promoted by numerous QS-orchestrated microbial interactions. These include collaborative or competitive relationships between *P. aeruginosa* and other species or strains of pathogens or with microbiota. (Figure 1).

### 2.1. Biofilm Development

Biofilms correspond to heterogeneous structures composed of bacterial microcolonies that are enfolded in an extracellular matrix fixed on an abiotic or biotic site. This self-produced matrix is essentially composed of water and polymeric substances, including exopolysaccharides (EPS) such as alginates, allowing adhesion to the tracheobronchial mucosa and two aggregative polysaccharides named Psl and Pel [3], proteins such as adhesins CdrA that establish CdrA–Psl and CdrA–CdrA interactions, promoting biofilm formation and stabilization [10], and high molecular weight extracellular DNA (eDNA), reinforcing the scaffolding of the protective barrier against the host immune system and antimicrobial agents [11,12].

Various strains or species of bacteria coexist inside biofilms thanks to their complementary metabolic profiles [13]. Biofilm formation is divided into five steps: attachment, cell-cell adhesion, proliferation, maturation and dispersion (Figure 1). First, reversible physicochemical interactions allow planktonic bacteria to attach on a surface. After that, the multiplication of micro-organisms induces an irreversible adhesion to the substratum, especially ensured by outer membrane LPS molecules. The third step corresponds to the formation of microcolonies composed of external bacteria joining the initial bacteria that have proliferated. Then, the progressive structuration of the biofilm matrix around the microcolonies under development leads to its maturation. The digging of canals occurs, allowing especially for the circulation of nutrients. Finally, some bacteria break away from the biofilm to disperse and promote the colonization of new sites [14]. Biofilm development plays an important role in the transmission of exogenous and endogenous nosocomial infections, providing to pathogens the ability to persist on abiotic surfaces, such as in healthcare settings, or biotic substrates, such as weakened tissues. After external host contamination or internal bacterial dissemination due to an invasive procedure, the establishment of new tissue reservoirs of persistent bacteria compromises wound healing or causes extensive microbial invasions.

Biofilms contribute to the virulence of *P. aeruginosa,* ensuring the colonization of host tissues, its immune escape and its resistance to harsh surroundings. The biofilm matrix acts as a natural barrier against the host immune system, preventing the bacterial antigen recognition by host antibodies and the penetration of phagocytic cells, such as granulocytes and monocytes. Furthermore, the phagocytosis process is blocked by the very large size of biofilms that appear slightly sensible to enzymatic lysis [15].

Biofilms also play a crucial role in anti-bioresistance. Firstly, the low diffusion of ATBs within the extracellular matrix reduces their ability to reach bacterial cells. The sessile biomass thus tolerates concentrations of ATB 1000 times higher than those normally required to kill planktonic germs [16]. Secondly, the poor availability of nutrients and the establishment of oxygen gradients inside biofilms lead to the reduction of cell metabolic activity and the appearance of dormant bacteria. These are more resistant to acute anti-infective treatments than growing cells and are especially responsible for biofilm ATB tolerance [17]. In addition, the spatial proximity between bacteria imposed by biofilms favours horizontal gene transfers, inducing the acquisition of resistances [18].

### 2.2. Virulence Factor Secretion

*P. aeruginosa* produces various virulence factors implicated in the host infection process, such as soluble adhesins and lectins, but also different pro-biofilm molecules promoting the adhesion to mucous membranes, proteolytic enzymes and siderophores favouring the colonization of tissues, exotoxins, different exoenzymes released by sophisticated secretion systems, rhamnolipids, and hydrogen cyanide, ensuring bacterial persistence by countering the immune system. In addition to their contribution to bacterial adhesion on alveolar epithelial cells during biofilm development, outer membrane porins such as OprF, belonging to the OmpA protein family, mediate the externalization of some of these virulence factors, such as pyocyanin, elastase B and exotoxin A [3]. Furthermore, *P. aeruginosa* also possesses five specific secretion systems. The types I and V (T1SS and T5SS) release into the extracellular environment toxins such as alkaline protease AprA for T1SS and adhesins CdrA for T5SS. Unlike the other systems, T5SS uses a two-step secretion mechanism involving a transport of the molecules through the periplasm. The most important secretion system, T3SS directly injects various exoenzymes (ExoU, ExoT, ExoS, and ExoY) into the cytoplasm of host immune system cells, leading to their death [3]. Finally, T2SS secretes various virulence factors such as exotoxin A, elastases A and B, type IV proteases and alkaline protease AprA [19].

#### 2.2.1. Proteolytic Enzymes

Several proteolytic enzymes, such as elastases, alkaline and type IV proteases, are produced by *P. aeruginosa* in order to colonize the host tissues and persist [3].

The elastase activity of *P. aeruginosa* plays an important role in its virulence and is mediated by the synergistic action of LasA and LasB. LasA is a serine protease that previously cleaves elastin of the host connective tissues to facilitate its final degradation by the zinc metalloprotease LasB. This lysis of elastin especially promotes invasion of the lung parenchyma by the pathogen. LasB is also able to hydrolyse other extracellular structural proteins such as collagen and fibrin as well as inactivate the immunoglobulins A and G (IgA and IgG) [8,20].

The alkaline protease AprA takes part in the cleavage of fibrin and elastin after its fragmentation by LasA. Furthermore, this enzyme slows chemotaxis of the neutrophil granulocytes down, allowing the bacteria to escape the phagocytosis [21,22].

The type IV proteases (PIV) promote immune evasion by degrading host IgG and contribute to tissue invasion and damage by compromising the structural integrity of various support proteins such as elastin. They are also involved in the lysis of host transferrins, enabling *P. aeruginosa* to pick up soluble iron via its own siderophores [23].

#### 2.2.2. Exotoxins

In order to kill host cells, *P. aeruginosa* uses its type II and III secretion systems (T2SS and T3SS) to secrete several exotoxins, such as exotoxin A, various exoenzymes and pyocyanin [3].

Exotoxin A (ETA) is the most toxic virulence factor secreted by *P. aeruginosa* via T2SS. This protein binds to host cells through CD91 or α2-macroglobulin transmembrane receptors, leading to its internalization by endocytosis. Then, ETA interrupts protein synthesis within the eukaryotic cells by inactivating the elongation factor 2 and induces necrosis. Finally, the damage tissue becomes a nutrient supply available for the bacteria [3,8,24].

The four exoenzymes ExoU, T, S and Y released via T3SS are implicated in host tissue invasion by *P. aeruginosa*. The phospholipidase A2 activity of ExoU allows the pathogen to irreversibly destroy the eukaryotic cell membrane, causing a rapid cell death. The adenylate cyclase effect of ExoY increases the intracellular level of cyclic nucleotides (cAMP, cCMP, cGMP and cUMP). This leads to a loss of the endothelial barrier integrity due to cell necrosis. ExoT is a bifunctional exotoxin. Its GTPase-activating and adenosine diphosphate ribosyl transferase activities synergistically contribute to impeding phagocytosis and disrupting epithelial barriers. ExoS exhibits a similar bifunctional profile and is involved in several stages of bacterial infection (colonization, invasion and dispersion) [3,25].

**Scheme 1:**
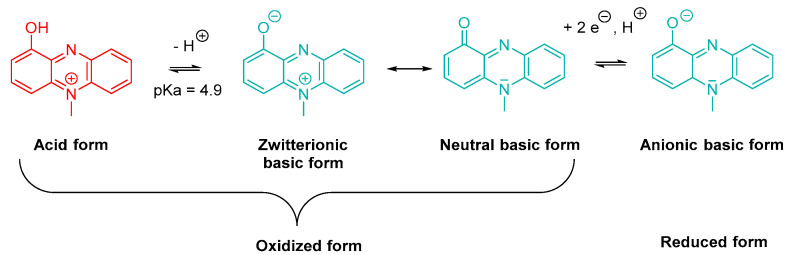
Oxidized and reduced (acid and basic) forms of pyocyanin.

Pyocyanin or 1-hydroxy-5-methylphenazine is a blue-green specific pigment produced by *P. aeruginosa*. Taking the weak acidic character of its phenol group (pKa = 4.9) into account, this exotoxin appears as a zwitterion at physiological pH (Scheme 1). Its basic neutral form is able to penetrate within cells by passive diffusion [26,27]. Furthermore, the molecule exhibits redox properties and also possesses a reduced basic form. Pyocyanin biosynthesis is divided in three steps, starting from chorismic acid [27,28].

Pyocyanin plays a crucial role in *P. aeruginosa* virulence. It induces oxidative stress in host tissue cells and exhibits a pro-inflammatory and immunosuppressive activity [8,26,29,30,31]. Under its oxidized form, this exotoxin is able to trap the electrons carried by NADPH and leads to an overproduction of ROS, such as superoxide ions and hydrogen peroxide [3,27,32]. A concomitant decrease in NADH concentration interrupts the mitochondrial respiratory chain and ATP synthesis. The resulting increase in intracellular dioxygen concentration accelerates the senescence process by exacerbating oxidative stress [3,27,32]. Pyocyanin also decreases the reducer to oxidant ratio of glutathione (GSH/GSSG) and inhibits superoxide dismutase (SOD) and catalase, inducing a failure of the antioxidant protective systems. [3,27,32]. A long-term exposure to pyocyanin encourages the occurrence of modifications in tracheobronchial cell gene expression, leading to an uncontrolled inflammatory response. Indeed, some of the overexpressed genes encode pro-inflammatory mediators that lure and activate the neutrophilic granulocytes, whose infiltration causes tissue damages [32]. The pyocyanin-induced oxidative stress stimulates the secretion of mucins, by tracheobronchial epithelial cells, leading to a mucociliary clearance slowdown and the establishment of a favourable environment for colonization. In addition, the ROS-induced cell lysis increases the eDNA release, contributing to the development of robust biofilms by *P. aeruginosa*. Finally, the leukopyocyanin resulting from the reduction of pyocyanin is involved in the acquisition of iron in oxygen-poor biofilms. Indeed, this colourless compound reduces Fe(III) from extracellular transferrins into Fe(II), which becomes available for bacterial internalization via Feo (Ferrous iron transport) system [33].

#### 2.2.3. Siderophores

In order to acquire the iron necessary for its growth, *P. aeruginosa* secretes two major siderophores, pyocheline and pyoverdine [34]. Pyoverdine chelates ferric iron with a stronger affinity than pyocheline (10^32^ M^−1^ vs. 10^28^ M^−1^, respectively) [35]. A total of three classes of pyoverdines were identified (PvdI, PvdII and PvdIII). These siderophores are able to chelate the Fe(III) dissolved in the external medium, but also to extract it from transferrins [36,37]. These hexadentate catechol-dihydroxamate type iron-carriers possess a dihydroquinoline chromophore linked to a peptide chain bearing complementary iron chelating groups [38]. The specific recognition of the Fe(III)/Pvd complex by the FpvA receptors of the outer membrane of *P. aeruginosa* depends on the different amino acid sequences defining the Pvd classes. Pyocheline, in the same manner as Pvds, plays an essential role during the host tissue colonization [39]. Furthermore, Pvds regulate the synthesis of other virulence factors such as ETA, type IV protease PrpL and FpvA receptor, and exert a positive feedback on their own production [8,28,40].

#### 2.2.4. Other Pro-Infectious Molecules

*P. aeruginosa* also secretes various other molecules, such as rhamnolipids, hydrogen cyanide and lectins, which are implicated in its pathogenicity.

##### Rhamnolipids

Rhamnolipids are extracellular amphiphilic glycolipids that are able to insert into biological membranes, owing to their surfactant properties, and are able to induce cell lysis. During the inflammatory reaction, the related destruction of phagocytes leads to the release of eDNA, useful for biofilm formation [15]. Furthermore, rhamnolipids accelerate at low concentrations LPS recruitment to the outer membrane, promoting bacterial adhesion on surfaces. However, the development of biofilms is impeded in case of surfactant overproduction [41].

##### Hydrogen Cyanide

Under low environmental dioxygen concentrations, *P. aeruginosa* synthesizes hydrogen cyanide via an enzymatic oxidative decarboxylation of glycine. This highly toxic molecule quickly diffuses through tissues and inhibits complex IV of the eukaryotic respiratory chain, leading to an oxidative stress exacerbation [42]. It is noteworthy that the pathogen has at its disposal a detoxification mechanism provided by the cyanide-insensitive terminal oxidase (CIO) [43,44,45].

##### Lectins

Lectins are soluble proteins involved in *P. aeruginosa* adhesion to host cells fastening on oligosaccharides of membrane glycoproteins (i.e., galactose for LecA and fucose for LecB), a LecA-promoted increase in the intestinal epithelium permeability to ETA and the structuring of the biofilm matrix, participating to the reticulation of EPS [46].

### 2.3. Host Tissue Colonization-Promoting Microbial Interactions

Bacteria are often found in multi-species microcolonies within biofilms, promoting various QS-regulated inter- and intra-species relationships. Most of these interactions are straightforward collaborations, such as the secretion of nutrient chelators, digestive enzymes, adhesins, matrix structural polymers and signalling molecules that benefit neighbouring cells. However, the microbial competition for space and limited resources is also widespread. Many social harmful phenotypes, such as the direct injection of toxins into adjacent cells or different stratagems to drive the opponents out or suffocate them, are described.

#### 2.3.1. Biofilm-Associated Collaboration

The elaborate biofilm matrix structuration allows the associated heterogenous biomass to have easier access to nutrients than planktonic bacteria since the proximity of the different species or strains favours exchanges. These exchanges allow the sharing as a courtesy of various colonization-promoting substances, such as proteolytic enzymes producing nutrients available for uptake by neighbouring cells from infected host tissues, biofilm matrix constitutive EPS, surfactants facilitating the bacterial mobility, siderophores or defensive antimicrobial molecules [13,47]. However, this inter- and intra-species cooperation potentially induces the emergence of cheaters trapping the public goods to their own advantage. An important increase in the cheater proportion in the bacterial population leads to an insufficient secretion of public goods and to a whole colony collapse. In other cases, the mutualism ensures a reciprocal benefit, such as when a waste product eliminated by a first species is used as a nutrient by a second one, thus purifying the environment [13].

#### 2.3.2. Intra- and Inter-Species Competition

In order to colonize the tissues, pathogens do not only have to defend themselves against the host immune system response. Indeed, competitive interactions also occur against other infectious agents and commensal bacteria of the microbiota living in protective symbiosis with the host. An important duel thus brings into opposition *P. aeruginosa* and *S. aureus* in polymicrobial infections. Mainly reported are two mechanisms: an indirect competition limiting the access to resources for the opponents and a direct competition leading to the death of less virulent strains or species owing to the release of different toxins [48]. 

##### Indirect Competition

Several mechanisms of indirect competition were described, such as appropriation of resources, inter-microbial censorship or favourable manipulation of the host immune system.

The nutrient-depleted environment inside biofilms promotes competition between micro-colonies for their survival. For example, *P. aeruginosa* expresses receptors able to recognize xenosiderophores produced by other pathogens at the expense of its competitors [49].

Furthermore, an inter-microbial censorship involving a degradation of signalling molecules synthesized by the opponents or a dispersion of their biofilm was evidenced. Various pathogens such as *P. aeruginosa*, *Streptomyces* sp. and *Agrobacterium tumefaciens* interfere with the QS of Gram-negative bacteria by degrading *N*-acyl-homoserine lactone (AHL)-type autoinducers using specific enzymes such as AHL oxidoreductases, lactonases or acylases [50]. Noteworthy, the plasma mammalian paraoxonases possess a similar AHL lactonase activity, participating to host protection against microbial infections [51]. Furthermore, AHLs act as repressors of the *S. aureus agr* (for accessory gene regulator) communication system. This leads to the inhibition of its secretion of exotoxins such as the fibronectin binding proteins implicated in the adhesion to host cells [52,53]. *P. aeruginosa* also produces a fatty acid, the *cis* 2-decenoic acid, able to induce a biofilm dispersion of various bacteria such as *S. aureus*, *E. coli* and *K. pneumoniae* [54].

In cystic fibrosis patients, *P. aeruginosa* is able to manipulate the host immune system to eradicate *S. aureus*. Indeed, the release of ExoS toxin induces the secretion of type IIA phospholipase A2 (sPLA2-IIA) by bronchial epithelium cells. This enzyme leads to a loss of integrity of the bacterial cell envelope via the hydrolysis of phosphatidylglycerol (PG) and phosphatidylethanolamine (PE) that are the main membrane phospholipids. Gram-negative bacteria are less sensitive to this attack than Gram-positive one because LPS molecules on the surface of their outer membrane prevent the penetration of sPLA2-IIA. This tactic is therefore deleterious for *S. aureus* [55]. Noteworthy, similar processes contribute to the synergistic defence of the host against pathogens by gut microbiota [56].

##### Direct Competition

The QS-controlled production of different antibacterial compounds such as bacteriocins or various respiratory chain inhibitors contributes to intra- and inter-species direct competition.

##### Bacteriocins

The bacteriocins are a heterogeneous group of anti-microbial peptides that mainly targeting Gram-positive bacteria. These toxins are helpful to the producing species in the invasion of already established microbial communities such as microbiota. However, the commensal bacteria also use them to prevent host colonization by pathogens. Among Gram-negative bacteria, 30–50% of *E. coli* and more than 90% of *P. aeruginosa* strains produce bacteriocins [57,58]. Pyocin S released by *P. aeruginosa* is a soluble bacteriocin able, in the same manner as colicin from *E. coli*, to form pores in the bacterial plasma membrane after binding to specific receptors. Owing to its additional nuclease activity, this antibacterial molecule finally causes cell lysis. Its activity is restricted to the intra-species competition between *P. aeruginosa* strains. On the contrary, pyocins R and F are described as phage tail protein scaffolds leading to membrane depolarization in different Gram-negative bacterial species [59].

##### Pro-Oxidative Toxins

In addition to its toxicity towards eukaryotic cells, pyocyanin also plays a role in the direct competition among *P. aeruginosa* and other bacterial species such as *S. aureus*. Its redox properties slow down the activity of the respiratory chain and provoke an energy depletion impacting the active transport systems [60,61]. Hydrogen cyanide produced by *P. aeruginosa* also inhibits the prokaryotic cell respiration and takes part in the duel against *S. aureus*.

*P. aeruginosa* produces various 2-alkyl-4-quinolone *N*-oxides (AQNOs) such as 2-heptyl and 2-nonyl-4-quinolone derivatives (HQNO and NQNO). HQNO, a secondary metabolite produced through its AQ biosynthesis pathway, appears as a potent anti-staphylococcal substance. This inhibits the prokaryotic and eukaryotic respiratory chain cytochrome *bc1* complex, causing ROS accumulation within the cell and leading to its apoptosis [62,63]. The conjugated action of HQNO and siderophores induces a metabolic transition from aerobic respiration to fermentation in *S. aureus*. This induces a production of lactates constituting a carbon source for *P. aeruginosa*. The combination of HQNO-induced fermentation and electron transport chain poisoning leads to the death of *S. aureus*. The resulting cell lysis allows the release of iron that becomes available for an uptake by *P. aeruginosa* [64].

## 3. QS Molecular Pathways in *P. aeruginosa*

**Figure 2:**
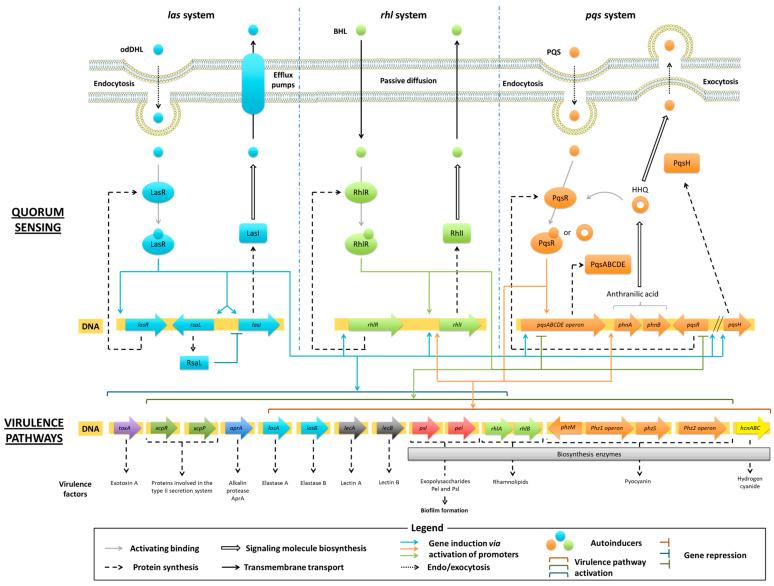
Interconnected QS networks of *P. aeruginosa* and regulation of virulence pathways.

Quorum Sensing (QS) of *P. aeruginosa* plays a crucial role in the regulation of virulence pathways during the infection process. QS relies on the secretion and the perception of small molecules called autoinducers (AIs). The concentration of AIs in the extracellular medium acts as a population density indicator. When the biomass grows, an increased release of AIs induces the expression of specific genes, ensuring the coordination of bacterial colonies regarding the environmental conditions. There were two main classes of QS systems described according to the nature of the secreted AIs and their signal transduction modes. On the one hand, Gram-negative bacteria usually produce *N*-acyl-homoserine lactones (AHLs) that spread into the cell and bind to a specific intracellular receptor protein. This leads to the activation of this transcriptional factor, regulating the expression of various genes, including those involved in the synthesis of AIs. On the other hand, Gram-positive bacteria synthesize autoinducing peptides (AIPs) that do not penetrate into the cell but bind to a specific transmembrane receptor and trigger a classical signal transduction pathway until the stimulation of targeted gene transcription [65]. QS of *P. aeruginosa* depends on three interconnected circuits named *las, rhl* and *pqs* systems (Figure 2). Whereas *pqs* system is the species-specific communication network, *las* and *rhl* systems using AHLs are found in other Gram-negative bacteria [66]. Through these different circuits, AIs regulate the expression of virulence pathways, exert a positive feedback on their own signalling pathway and exhibit modulatory properties on the other two systems [67].

### 3.1. Las and Rhl Systems

**Scheme 2:**
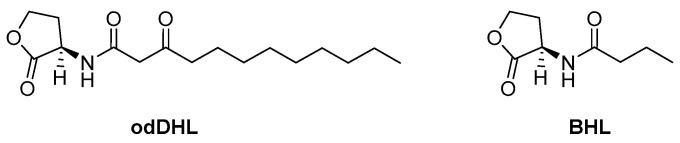
Respective AIs of *las* and *rhl* systems: the *N*-(3-oxododecanoyl)-L-homoserine lactone (odDHL) and the *N*-butanoyl-L-homoserine lactone (BHL).

The signal transmission by *las* and *rhl* circuits relies on the secretion of AHL-type AIs: the *N*-(3-oxododecanoyl)-L-homoserine lactone (odDHL) for *las* system and the *N*-butanoyl-L-homoserine lactone (BHL) for the *rhl* one (Scheme 2).

In *P. aeruginosa*, the biosynthesis of AHLs is associated to the fatty acid one. Following the initiation step, allowing the synthesis of acetyl-ACP (acyl carrier protein), repeated cycles of elongation ensure the production of acyl-ACP lipid precursors [68]. The LasI and RhlI enzymes catalyse the synthesis of odDHL and BHL from the obtained 3-oxo-acyl-ACP and crotonyl-ACP, respectively. Secreted AHLs are internalized by neighbouring bacteria via endocytosis for odDHL and passive diffusion for BHL, and bind to their corresponding cytoplasmic LasR or RhlR receptor protein. The resulting LasR(odDHL) or RhlR(BHL) complex induces the gene transcription of various virulence factors and specific proteins associated with the communication pathway. The LasR(odDHL) activated transcriptional factor up-regulates the expression of *lasI* and *lasR* genes encoding for LasI synthase and LasR receptor. The active RhlR(BHL) and LasR(odDHL) complexes induce the transcription of *rhlI* and *rhlR* genes encoding for RhlI synthase and RhlR receptor, respectively. The secretion of AHLs is finally ensured by ABC (ATP binding cassette) efflux pumps for odDHL and by passive diffusion for BHL.

The LasR(odDHL) complex appears as a *pan*-activator of the three communication *las*, *rhl* and *pqs* circuits, raising it to the top of the *P. aeruginosa* QS hierarchy. In order to control the production of AHLs, the *las* system is also down-regulated by a transcriptional repressor named RsaL, encoded by *rsaL* gene. The expression of these modulators is induced by the LasR(odDHL) complex. RsaL inhibits the *lasI* transcription owing to a simultaneous binding to the *lasI* promoter and the LasR(odDHL) complex. RsaL also decreases the production of pyocyanin and hydrogen cyanide, directly repressing the corresponding gene promoters [28]. 

Together, the *las* and *rhl* systems regulate about 300 genes in *P. aeruginosa,* corresponding to 4–12% of its genome and including the virulence ones. The *las* circuit stimulates the expression of *toxA* (exotoxin A), *xcpR* and *xcpP* (proteins involved in the type II secretion system), *aprA* (AprA alkalin protease), *lasB* and *lasA* (elastases A and B), *lecA* and *lecB* (lectins A and B) genes, but also *psl* and *pel* (Psl and Pel exopolysaccharides) ones [69]. In addition to exacerbating these virulence pathways, the *rhl* circuit also induces the secretion of rhamnolipids by activating the transcription of *rhlAB* gene [67,70,71,72]. 

### 3.2. Pqs System

**Scheme 3:**
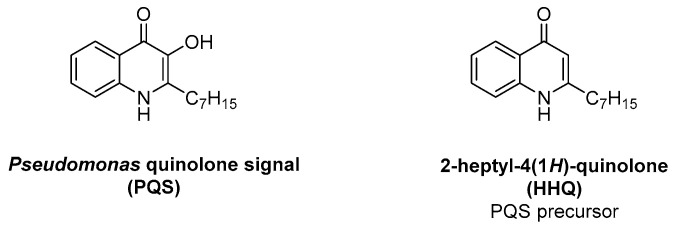
AIs of *pqs* system: the 2-heptyl-3-hydroxy-4(1*H*)-quinolone (PQS) and the 2-heptyl-4(1*H*)-quinolone (HHQ).

*P. aeruginosa* bacteria have a third specific communication network called *pqs* system that is based on 2-alkyl-4(1*H*)-quinolone (AQ) signalling molecules. The main AI of this circuit is the 2-heptyl-3-hydroxy-4(1*H*)-quinolone, named *Pseudomonas* quinolone signal (PQS), eventually assisted by its precursor, the 2-heptyl-4(1*H*)-quinolone (HHQ) (Scheme 3).

**Scheme 4:**
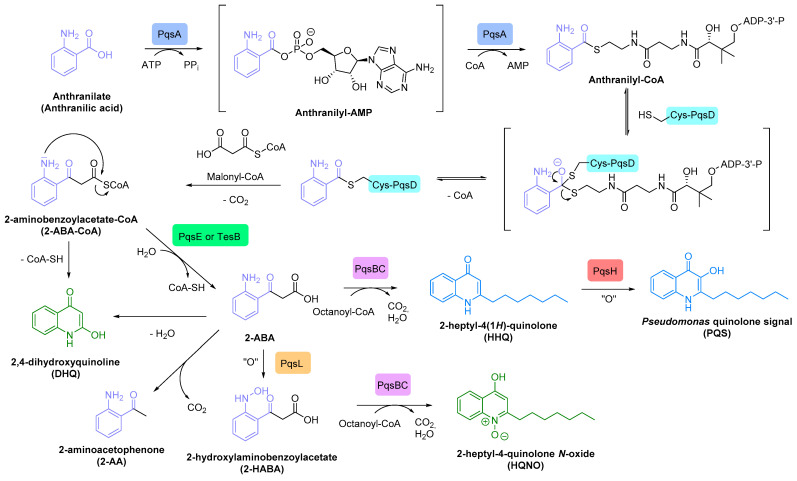
AQ biosynthesis with signalling molecules in blue and toxic secondary metabolites in green.

Once internalized by neighbouring bacteria via endocytosis, PQS synthesized by *P. aeruginosa* binds to its PqsR receptor, also known as the multiple virulence-factor regulator (MvfR). High cytoplasmic concentration of this activated PqsR (PQS) transcriptional regulator triggers the expression of *pqsABCDE* operon and leads to the production of PqsA, B, C, D and E enzymes involved in the AQ biosynthesis. The PqsR (PQS) complex also induces the transcription of *phnA* and *phnB* genes, encoding for synthases implicated in the production of anthranilate, the starting substrate of the HHQ/PQS metabolic pathway. The final conversion of HHQ into PQS is catalysed by the PqsH monooxygenase. PQS is then released in the extracellular medium by exocytosis (Scheme 4) [70,73]. Noteworthy, the TesB thioesterase is able to replace the PqsE enzyme in its metabolic functions [74]. Through this pathway, *P. aeruginosa* produces more than 50 other AQs, such as HQNO and DHQ secondary metabolites, displaying a prokaryotic and eukaryotic toxicity, respectively.

The *pqs* circuit essentially stimulates the secretion of pyocyanin, but also the production of elastases A and B, outer membrane vesicles (OMV) involved in the transport of PQS, lectins A and B, hydrogen cyanide, efflux pumps and various virulence factors implicated in the biofilm formation, such as rhamnolipids and exopolysaccharides (Pel and Psl) [73,75]. It also up-regulates the *rhl* circuit by inducing *rhlI* expression [76]. The RhlR(BHL) complex down-regulates the expression of *pqsR* gene, whereas the LasR(odDHL) transcriptional factor up-regulates it, as well as the *pqsH* one [77].

Given the important role of QS in *P. aeruginosa* pathogenicity, the interest of these communication systems as reservoirs of promising therapeutic targets for the development of inhibitors has emerged in the recent decades to fight against anti-bioresistance.

## 4. *P. aeruginosa* QS-Quenching Anti-Virulence Agents

The QS inhibition, called Quorum Quenching (QQ), is already used by micro-organisms competing for the same ecological niche. For example, the signal molecule odDHL secreted by *P. aeruginosa* appears to be a gene repressor of the QS in *S. aureus* [52]. Thus, various QQ methods are known in bacterial colonies, such as the production of QS inhibitors (QSIs) blocking the self-induction or the biosynthesis of signal molecules or the secretion of enzymes able to denature extracellular AIs [78]. Considering the development of AVAs, three QQ strategies mimicking these bacterial mechanisms have emerged: the design of antagonists of AI receptors or inhibitors of the enzymes involved in their biosynthesis, the capture of AIs by antibodies or other macromolecules such as cyclodextrins and the enzymatic degradation of extracellular AIs, especially AHLs, using lactonases, phosphotriesterase-like lactonases (PLLs), acylases or oxydoreductases [78,79].

In this review, we will focus on the different advances in the development of selective QSIs against *P. aeruginosa* through a ligand-based strategy. Considering the QS sophisticated network regulating the pathogenicity of *P. aeruginosa*, three major quorum silencing pharmacological approaches emerge, including the identification of LasR-targeting *pan*-QSIs, RhlR-modulating QSIs, and PqsR and AQ synthase-targeting QSIs. Given the widespread occurrence of AHL-mediated communication systems in the microbe kingdom, a lot of quorum quenchers are secreted by various plants, fungi or animals to protect themselves against bacterial colonization [80]. These naturally-occurring molecules will not be herein studied.

### 4.1. LasR-Targeting Pan-QSIs

The las system regulates both rhl and pqs, via the activated transcription factor LasR(odDHL), placing it at the top of the QS hierarchy in *P. aeruginosa*. The LasR receptor thus appears as a key target for the development of pan-QS inhibitors, leading to a hasty interruption of the communication cascade and a drastic decrease in the pathogen virulence. Interestingly, such quenchers often additionally afford a synergistic dual-acting profile, disrupting AHL autoinduction of both las and rhl circuits via a binding on their respective transcription factors.

#### 4.1.1. AHL Autoinducer Analog QSIs

**Scheme 5:**
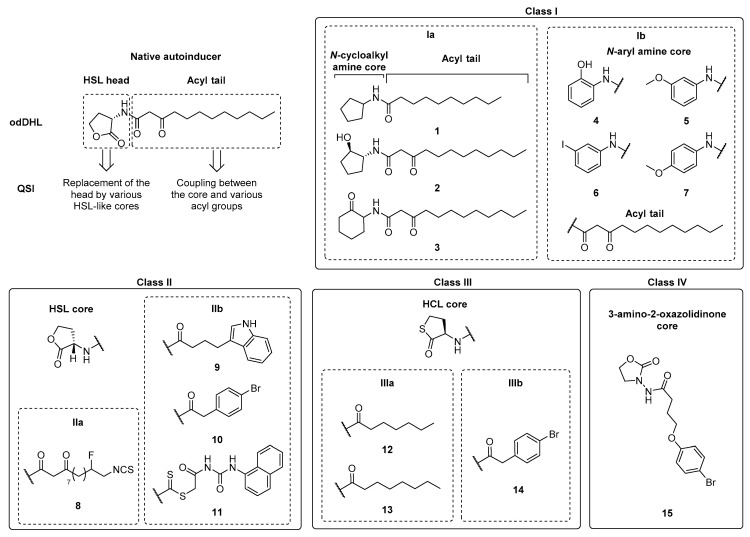
Classification of synthetic AHL autoinducer analog QSIs.

The modification of the HSL head or the acyl tail of native AHL autoinducers provides four classes of synthetic competitive LasR/RhlR inhibitors possessing: a *N*-cycloalkyl or *N*-aryl amine (**class Ia-b**), a HSL (HomoSerine Lactone) (**class II**), a HCL (HomoCysteine Lactone) (**class III**) or a 3-amino-2-oxazolidinone (**class IV**) core acylated with various groups (Scheme 5, Table 1).

Ishida et al. synthesized a series of *N*-acyl cyclopentylamide analogs belonging to the **class Ia**. The *N*-decanoyl derivative **1** demonstrated the stronger QQ activity and the ability to inhibit LasR(odDHL) and RhlR(BHL) interactions (Scheme 5, Table 1) [81]. This inhibition is dependent of the acyl side chain length (50% of LasR inhibition for the *N*-octanoyl analog at 250 µM vs. 80% for **1**). Smith et al. studied the structure–activity relationships of different odDHL analogs as LasR and RhlR agonists or antagonists [82,83]. In their library, the HSL moiety was replaced by various cycloalkyl, aryl or heteroaryl amines. Whereas the *N*-(2-hydroxycyclohexyl)-3-oxododecanamide was revealed as a potent agonist, the *N*-(2-hydroxycyclopentyl), *N*-(2-oxocyclohexyl) and *N*-(2-hydroxyphenyl) derivatives **2**, **3** and **4** appeared as promising LasR antagonists (Scheme 5, Table 1). The presence of a hydrogen bond acceptor (keto or hydroxyl group) adjacent to the acylated amine function in a five- or six-membered ring is required for the binding to LasR. Furthermore, the comparison of the *N*-(2-hydroxycyclohexyl) agonist with its *N*-(2-hydroxyphenyl) analog **4** showed that the replacement of a saturated ring by an aromatic one allowed to switch an agonist for an antagonist. Interestingly, the compound **3** was found to be the most potent inhibitor of biofilm, pyocyanin and elastase expression. This could be due to its ability to inhibit both LasR(odDHL) and RhlR(BHL) interactions (35% and 60% of inhibition at 100 and 50 µM in *lasI-gfp* and *rhlI-gfp* reporter gene assays in a *P. aeruginosa* PAO-JP2 strain). Indeed, the native odDHL and BHL are known as RhlR antagonist and agonist, respectively, demonstrating that this receptor could allow the fixation of derivatives bearing an acyl chain of 4 or 12 carbons. It was observed that analogs of **2** and **4** that only possess a four carbon chain are not able to activate RhlR on the contrary of the analog of **3**. Finally, in 2015, Moore et al. redefined the compounds **3** and **4** as partial agonists of LasR [84]. In the **class Ib**, the strong anti-pyocyanin activity of three AHL mimics the non-native aromatic head core described by Hodgkinson et al. (93% of inhibition for **5**, 67% for **6** and 73% for **7** at 200 µM), confirming the importance of a hydrogen bond acceptor on this head group for LasR interaction, in addition to the presence of a long acyl chain (Scheme 5, Table 1) [85].

In the **class II** of AHL analogs bearing a HSL core and a modified acyl chain, the introduction of an isocyanate group by Amara et al. provided an irreversible covalent binding with the SH group of the Cys79 of LasR, causing the receptor inactivation. The compound **8** (**class IIa**, IC_50_ of 154 μM in a *P. aeruginosa* PAO-JP2 strain) possesses a stronger affinity than its non-halogenated analog in the autoinduction pocket (Scheme 5, Table 1) [86]. It allows to improve the clearance of *P. aeruginosa* (PA14) in both bacterial infection models studied (*C. elegans* and ex vivo-grown human burn wound skin samples). Geske et al. introduced various aromatic groups on the AHL scaffold (**class IIb**) and showed that the indolyl-AHL **9** and the bromophenyl-AHL **10** were 2-fold more efficient QSIs than compound **4,** described by Smith et al. (Scheme 5, Table 1) [80]. In 2015, Moore et al. demonstrated that the derivative **10** is a partial agonist of LasR [84]. More recently, Liu et al. focused on the structure–activity relationships of novel AHL analogs that possess a specific linker between the core and the lateral acyl group [89]. Among them, the phenylurea-containing *N*-dithiocarbamated HSL **11** demonstrated the best activity and inhibits the expression of virulence factors such as pyocyanin, elastases and rhamnolipids, but also biofilm formation at micromolar concentrations (Scheme 5, Table 1). A molecular modelling study displayed that **11** was the only QSI among these AHL analogs to interact by hydrogen bonds with the Ser-129 and Arg-61 key residues located in the autoinduction site of LasR.

The **class III** is represented by thiolactone analogs of AHLs possessing a HCL core acylated with an alkyl saturated chain (**class IIIa**, QSIs **12** and **13**) or an aromatic hydrophobic substituent (**class IIIb**, QSI **14**) [90]. These compounds showed antagonist activity in an *E. coli* LasR reporter gene assay with IC_50_s values of 0.79, 0.14 and 0.40 µM, respectively. However, they had no antagonist effects in a *P. aeruginosa* LasR reporter gene assay (Scheme 5, Table 1). In the **class IIIa**, the length of the acyl chain is important for the selectivity towards the receptor (LasR vs. RhlR). Interestingly, the substitution of a HSL core by a HCL one (**10** vs. **14**) induced a loss of the QQ efficiency. Noteworthy, McInnis et al. reported two possible operating mechanisms of LasR transcription factor dimers: the “cooperative agonist” mode in which non-native ligands are able to form homodimers, ensuring an inhibition, but also active heterodimers with odDHL, and the “bimodal ligand” mode, in which the heterodimers are inactive.

Jiang et al. recently synthesized and evaluated the potential of 3-amino-2-oxazolidinone AHL analogs as QSIs that belong to the **class IV**. The most active derivative **15** exhibited interesting anti-virulence properties against a *P. aeruginosa* PAO1 strain at 162.5 µM, with 40, 22, 16 and 57% of inhibition of biofilm, pyocyanin, elastase and rhamnolipid production, respectively (Scheme 5, Table 1). This compound prolonged the lifespan of PAO1-infected nematodes in combination with meropenem, enhancing the sensibility of biofilm bacteria to the ATB [91].

To conclude, the design of AHL analogs to inhibit LasR is a promising strategy, but one that could be difficult to apply. Indeed, slight modifications of the antagonist core mimicking the HSL head potentially lead to an agonist activity or inactivity. This is due to the high affinity and specificity of odDHL to its receptor. However, the replacement of the HSL head by a *N*-cycloalkyl or *N*-aryl amine or HCL core confers to the QSIs a better stability against hydrolysis.

#### 4.1.2. AHL Autoinducer Non-Analog QSIs

**Scheme 6:**
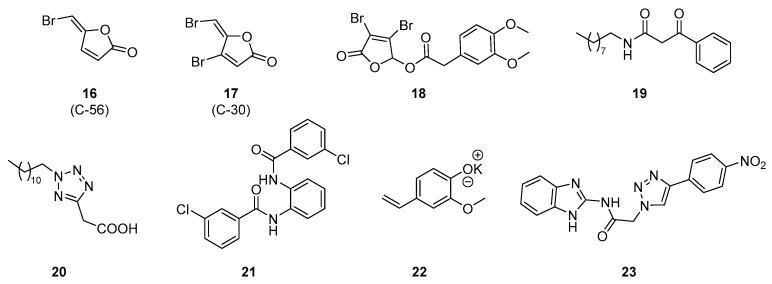
AHL autoinducer non-analog QSIs.

Hentzer et al. synthetized various synthetic furanones as analogs of secondary metabolites produced by the Australian marine red macroalga *Delisea pulchra* [92]. These metabolites are halogenated furanones that possess strong bacterial properties and some of them show AHL antagonistic activity. Although natural furanones have limited effects on *P. aeruginosa* QS, the halogenated furanone **16** (C56) is able to decrease the expression of *lasB* regulated by *lasR* in a reporter gene assay and the extracellular elastase and chitinase activities (Scheme 6, Table 2). This derivative could even interfere with the QS of *P. aeruginosa* in the biofilm matrix and inhibit biofilm maturation. In vivo evaluations were carried out in *P. aeruginosa* lung infection mice models. Both compounds **16** and **17** (C30) improved the clearance of bacteria from the lung, impaired that colonization, reduced the severity of pathology and significantly prolonged the mice’s survival time [93]. The mechanism of action of these compounds is still unknown but a supplementary interaction with the *rhl* system was supposed. Noteworthy, Moore et al. recently demonstrated that compound **17** has no activity on the LasR receptor [84]. Recently, Chang et al. synthetized novel promising synthetic furanones bearing alkyl chains or aromatic groups. Among them, the lead compound **18** inhibits the biofilm formation and reduces the pyocyanin production by potentially interfering with LasR (Scheme 6, Table 2) [94]. It showed weak cytotoxicity and promising ADME properties for a future development.

Müh et al. carried out a high-throughput cell-based screening of a library of approximately 200,000 products [95]. A total of two synthetic phenacyle and 2-alkyltetrazole compounds **19** and **20** were revealed as efficient LasR inhibitors in a *P. aeruginosa* PAO-MW1 strain, deficient in acyl-HSL synthases LasI and RhlI, with IC_50_s of 10 µM and 30 nM, respectively (Scheme 6, Table 2). They affected the QS-dependent production of virulence factors (pyocyanin and elastases) by potentially binding to LasR, owing to their structural similarity to the native signalling molecule odDHL. The same team also described synthetic triphenyl derivatives able to interfere with the QS [96]. Compound **21** is a LasR antagonist with an IC_50_ of 50 µM (Scheme 6, Table 2). In silico docking studies of **21** in the LasR binding pocket showed that the chlorine substituent of the antagonist derivative interacts with the Asp73 residue. However, an activating interaction implicates a supplementary bond with the Trp60 from a secondary hydrogen bond acceptor group such as in odDHL. Furthermore, this compound **21** possesses a better stability in alkaline media but also against enzymatic degradation than odDHL. Moore et al. recently confirmed the antagonist activity of compounds **19**, **20** and **21** in a *P. aeruginosa* PAO-JP2 strain.

Shah et al. previously synthetized a 2-methoxy-4-vinylphenolate derivative with a significant activity as QSI. Its aqueous solubility was greatly improved, owing to the formation of a potassium salt **22** that thus exhibited an increased anti-QS efficiency. This compound demonstrated various anti-virulence properties in *P. aeruginosa,* such as a reduction of production of LasA and LasB proteases, but also pyocyanin, biofilm formation and bacterial motility by targeting *las* and *rhl* circuits (Scheme 6, Table 2) [97].

Srinivasarao et al. screened a series of triazole–benzimidazole hybrids for their anti-QS activities [98]. As described by Blackwell et al., the introduction of a 1,2,3-triazole group on the acyl chain of odDHL analogs provided LasR antagonist activity. The lead compound **23** showed a good efficiency as QSI with 64% inhibition at 62.5 µM in a *P. aeruginosa* MH602 *lasB* reporter strain assay (Scheme 6, Table 2). Docking studies highlighted that it could establish π–π stacking interactions in the LasR autoinduction site. The substitution of the 1,2,3-triazole core with a phenyl ring bearing an electron-withdrawing group at *para* position greatly increased the QSI activity.

Most of the described LasR inhibitors present inconveniences, such as the presence of sensitive cores to enzymatic degradation, such as thiolactone or lactone rings, a strong lipophilicity or molecular weight, or both, or an ambivalent anti-virulence profile against *P. aeruginosa* clinical strains. Noteworthy, the study of Moore et al. allowed an interesting comparison of LasR inhibitory activities of compounds **1**, **3**–**5**, **10**, **17** and **19**–**21** (reporter gene assay on *P. aeruginosa* (PAO-JP2, p*lasI*-LVA*gfp*) with competition against odDHL (150 nM)). To the best of our knowledge, none of the developed *pan*-QSIs have successfully passed the preclinic development phase.

### 4.2. RhlR-Modulating QSIs

The virulence regulation by the *rhl* circuit is very subtle, due, in particular, to the repressive control exerted by RhlR on the *pqs* system. The development of AVAs targeting RhlR thus involves the design of negative modulators to turn off the *rhl* system or positive ones to inhibit the *pqs* circuit. Keeping in mind that in the case of antagonists, the effect on virulence is strongly dependent on their affinity for the target. Considering the virulence of *rhlI*-deficient mutants not being impacted in murine model infections, the interest of RhlR as an anti-QS pharmacological target was initially questionable. Thus, only a few compounds have so far been described as RhlR modulators [99].

**Scheme 7:**
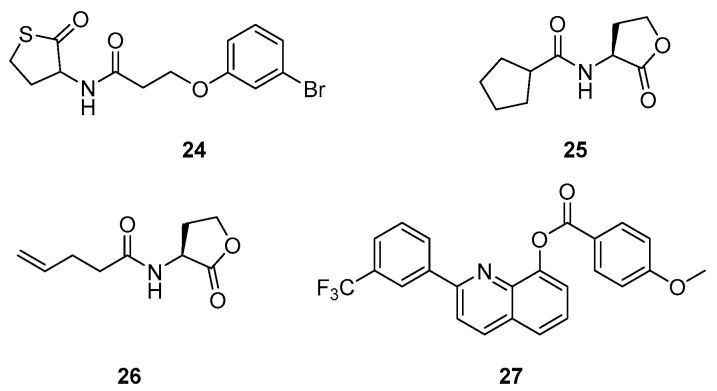
RhlR-modulating QSIs.

Compound **24** showed RhlR agonist properties, reducing pyocyanin production (IC_50_ of 8 µM, *P. aeruginosa* PA14 strain) due to repression of the *pqs* circuit (Scheme 7, Table 3). It also inhibits biofilm formation and improves survival of *C. elegans* at 50 µM during a *P. aeruginosa* infection [100]. In 2015, Moore et al. also described **24** as a partial agonist of LasR [84]. Likewise, BHL analogs **25** and **26** significantly inhibit pyocyanin production as RhlR agonists in a *lasI-gpf* reporter gene assay in a *P. aeruginosa* PAO-JP2 strain with EC_50_s of 4.5 and 7.2 µM, respectively (Scheme 7, Table 3) [101]. However, this activation of RhlR potentially leads to an overproduction of rhamnolipids involved in bacterial mobility, biofilm maturation and dispersion, and therefore facilitates the infiltration of *P. aeruginosa* within the bronchial epithelium [102,103,104]. Nevertheless, Qiu et al. recently synthesized various specific antagonists of RhlR that possess a quinolin-8-ol structure. The lead compound **27** inhibits the rhamnolipid and biofilm production (40 and 15% at 10 µM, respectively) without affecting the pyocyanin secretion (Scheme 7, Table 3) [105].

Modulation of the RhlR receptor could constitute an interesting approach because of its particular role in the bacterial communication network. For this reason, in-depth studies are currently underway. However, due to the fact that the AHL-dependent communication systems are widespread in the bacterial kingdom, the inhibition of *las* and *rhl* circuits could have the disadvantage of affecting the host microbiota. On the contrary, the pqs circuit based on 2-alkyl-4(1H)-quinolone signalling molecules offers the possibility to design pharmacological tools specifically directed against P. aeruginosa.

### 4.3. PqsR and AQ Synthase-Targeting QSIs

A total of two interesting strategies to block the pqs circuit have been described, including the inhibition of the PqsR receptor by competing against the binding of its native autoinducers, HHQ and PQS, and the metabolic biosynthesis pathways of HHQ and PQS by targeting various enzymes, such as PqsA, PqsD, and PqsBC.

#### 4.3.1. PqsR Inhibitors

PqsR is the most studied target of the *pqs* circuit for the development of AVAs due to its crucial position at the beginning of the activation circuit.

##### AQ Autoinducer Analog QSIs

**Scheme 8:**
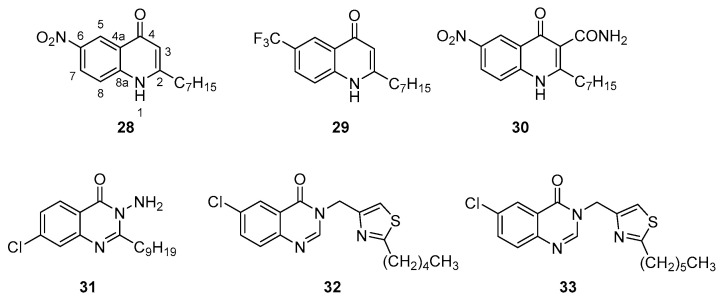
AQ autoinducer analog QSIs possessing a quinolone or quinazolone core.

Hartmann’s team discovered PqsR antagonists by performing various pharmacomodulations on the quinolone core of HHQ. Compounds with an electron withdrawing group at position 6 of the quinolone core present the best anti-QS potential in an *E. coli* reporter gene assay (**28** and **29**, IC_50_s of 51 and 54 nM, respectively) (Scheme 8, Table 4) [106]. However, they only induce a weak reduction in the secretion of pyocyanin in *P. aeruginosa* and do not affect the production of elastases, rhamnolipids and AQs. It appeared that the position 3 of the quinolone core could be hydroxylated by the PqsH enzyme, thus converting these antagonists into agonists. On the contrary, the derivative **30** with a carboxamide group in position 3 preserved its PqsR antagonist activity in *P. aeruginosa* (IC_50_ of 1.3 μM) and efficiently reduced the pyocyanin production with an IC_50_ of 1.9 µM (Scheme 8, Table 4) [107]. In vivo assays were carried out in two animal infection models. This compound is able to protect the host from *P. aeruginosa* infections in both nematode and insect model experiments [108]. In 2017, the compound **30** was requalified as an inverse agonist of PqsR because it does not only suppress the autoinduction of the receptor, but also its basal effect, corresponding to its constitutive activation in the absence of AIs. Thus, it induces a conformational change of the receptor causing a strong inhibition. Docking studies demonstrated that several interactions are involved in its fixation inside the autoinduction site of PqsR: hydrophobic interactions between the heptyl chain and the Ile186, Leu189 and Tyr258 amino acids, π-σ interactions between the quinolone core and the Ile236 and Ile186 residues, a hydrogen bond between the nitro group and Ile236 via a water molecule and a hydrogen bond between the carboxamide substituent in position 3 and the carbonyl acceptor group of Leu207 and Arg209, causing a conformation change of the receptor by removing the basal engagement of a water molecule with the protein backbone. Taking into account the participation of the hydroxyl group in position 3 of PQS as an acceptor in a hydrogen bond with Leu208 and Arg209 via a water molecule, its replacement by a hydrogen bond donor group in compound **30** thus allows to switch from an agonist to an inverse agonist.

Ilangovan et al. studied the interactions of HHQ, PQS and other 2-alkyl-quinolone molecules with PqsR [109]. Their agonist or antagonist profile and their binding modes to the receptor were compared with those of various newly synthesized 2-alkyl-quinazolone analogs. Among these novel compounds, three 3-amino derivatives bearing an electron-withdrawing group in position 6 or 7, or both, appeared as interesting inhibitors of PqsR (IC_50_s for 7-fluoro, 6,7-difluoro and 7-chloro-3-amino-2-nonyl-quinazolones of 3.9, 1.2 and 5 µM in a *P. aeruginosa* reporter gene assay, respectively). In the quinazolone series, the replacement of the hydroxyl group in position 3 of PQS by a primary amine function switches the agonist activity into an antagonist one. The compound **31** exhibits a nearly IC_50_s of 50 µM against pyocyanin production and an ability to limit the development of biofilms in *P. aeruginosa* PAO1 strain (Scheme 8, Table 4). Docking studies demonstrated that this promising AVA is fastened to the PqsR autoinduction pocket as a competitive inhibitor. The formation of a halogen bond (assimilated to a hydrogen bond) between its 7-chloro substituent and the side chain hydroxyl group of Thr265 was highlighted. Furthermore, the activation of the protein is hindered by the interaction of the hydrogen bond donor amine function in position 3 of **31** with the Leu207 as for the QSI **30** described by Hartmann et al., and a subtle difference of orientation of the quinazolone core in the binding site compared with quinolone one of AIs.

Grossman et al. developed new 3-thiazolomethyl-quinazolone analogs, with a strong inhibitory potential of PqsR [110]. The derivatives **32** and **33** exhibit good anti-QS activities (IC_50_s of 313 and 342 nM for **32** and 289 and 265 nM for **33** in PAO1-L and PA14 strains, respectively) and reduce the pyocyanin production in a PAO1-L strain (23 and 36% inhibition at 939 and 867 nM for **32** and **33**, respectively) (Scheme 6, Table 4). Docking studies suggested that an interaction via a hydrogen bond is possible between the carbonyl function at position 4 of the quinazolone ring and the hydroxyl group of Thr265 as for the 2-alkyl-quinazolone derivative **31**. In 2021, Grossman’s group described a new quinazolone derivative as a PqsR antagonist (IC_50_ of 1.1 µM in a *P. aeruginosa* PAO1-L strain) with an anti-pyocyanin activity similar to compounds **32** and **33** (80% of inhibition at 3 µM in a *P. aeruginosa* PAO1-L strain) [111].

**Scheme 9:**
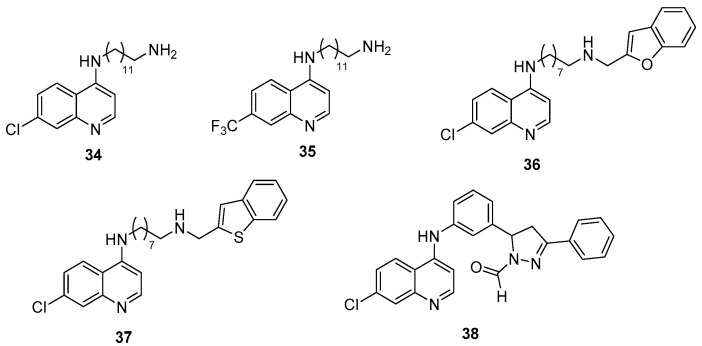
AQ autoinducer analog QSIs possessing a 4-aminoquinoline core.

A total of two series of 7-substituted 4-(alkylamino)quinolines were developed by Aleksić et al. with an amine function in position 4 substituted by a long primary aminoalkyl chain (**34** and **35**) or a long *N*-substituted aminoalkyl chain (**36** and **37**) [112,113]. A total of three 7-chloro (**33**, **35** and **36**) and one 7-trifluoromethyl (**34**) derivatives exhibited promising results as anti-virulence agents in *P. aeruginosa* by acting on the reduction of pyocyanin secretion (IC_50_s of 140, 2.5, 12 and 25 µM for **34**, **35**, **36** and **37**, respectively), biofilm formation (40 and 50% of inhibition at 140 µM and 125 µM for **34** and **35** and IC_50_ of 50 µM for **36**, respectively), bacterial motility (by 40 to 60% at 50 µM for **36** and **37**) and elastase production (35 and 30% of inhibition at 50 µM for **36** and **37**, respectively) (Scheme 9, Table 4). Noteworthy, the 7-trifluoromethyl derivative **35** shows similar anti-pyocyanin activity as the 2-alkylquinolone derivative **30**, previously described by Hartmann et al. It is also 20-fold more potent than the 2-alkylquinazolone derivative **31**, described by Ilangovan et al. These derivatives also presented anti-QS activity with inhibition of PQS production (85% of inhibition at 140 µM for **34**, 75% at 125 µM for **36**, 60% at 50 µM for **37** and 40% at 50 µM for **35**). The PqsR antagonistic activity of compounds **36** and **37** (IC_50_s of 25 and 30 µM, respectively) has a direct phenotypical impact on pyocyanin production. Structure–activity and structure–property relationship studies suggest that the moderate lipophilicity of active derivatives facilitates the transport requirement through cell membranes. In the autoinduction site of PqsR, the 4-(alkylamino)quinolones compete with Ais, especially for the crucial hydrogen bond formation with the Leu207 residue. The importance of the quinoline core substitution in position 7 by a hydrogen bond acceptor group was again highlighted in this QSI family. Furthermore, the antagonists **36** and **37** could engage additional interactions with PqsR through their *N*-substituted aminoalkyl chain, including hydrophobic bonds through the alkyl chain, hydrogen or ionic bridges from their 4-amino function and π-stacking through their benzofuran or benzothiophene ring.

At the same time, the interest of the 4-aminoquinoline core for the design of new PqsR inhibitors was confirmed by Soukarieh et al. by the identification of the 4-(arylamino)quinoline derivative **38** (IC_50_s of 2.3 and 12.4 µM in PA14 and PAO1 strains, respectively) (Scheme 9, Table 4) [114]. This compound exhibits promising anti-QS, anti-pyocyanin and anti-biofilm properties in different *P. aeruginosa* strains (PAO1 and PA14) without affecting bacterial growth. In combination with tobramycin, it could restore the ATB sensibility of these micro-organisms by disrupting the integrity of the protective biofilm. Docking studies showed that these 4-aminoquinoline QSIs adopt similar conformations to the reported 2-alkylquinazolones in the autoinduction pocket. In fact, the 4-amino function of the quinoline core also interacts with the Leu207 but via electrostatic interactions. Furthermore, the essential role of the chlorine or trifluoromethane group at the 7-position for the fixation towards the Thr265 amino acid is confirmed.

**Scheme 10:**
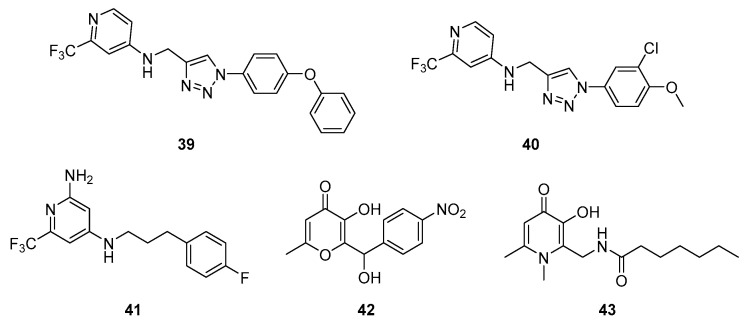
AQ autoinducer analog QSIs possessing a pyridine, pyranone or pyridinone core.

In 2019, Hartmann’s group patented 4-aminopyridineAQ analogs as PqsR antagonists. The most active derivatives were **39** and **40** with IC_50_s ≤ 50 nM in a PqsR *E. coli* reporter gene assay (Scheme 10, Table 4) [115]. These two compounds demonstrated interesting anti-pyocyanin (IC_50_s ≤ 250 nM and 250–1000 nM for **39** and **40** in a breathable seal PA14 assay, respectively) and anti-biofilm properties (IC_50_s ≤ 500 nM for **39** and **40** towards biofilm formation in PA14, respectively) without affecting bacterial growth. During hepatic metabolic stability assays, they presented a half-life time between 41 and 60 min. Furthermore, the compound **40** revealed less cytotoxicity than its analog **39** (IC_50_ ≥ 50 µM vs. 25–50 µM towards human HepG2 cells). This research team also studied the anti-QS properties of the 4-aminopyridine derivative **41** that possesses an IC_50_ of 0.14 µM in an *E. coli* reporter gene assay [116]. It showed a good anti-pyocyanin activity with an IC_50_ of 5.9 µM. The introduction of a suitable size flexible linker between the pyridine core and the phenyl group allows an optimal conformation of **41** in the autoinduction site of PqsR. This compound is able to compete with native PqsR ligands. The pyridine core is positioned into the HHQ quinolone core pocket whilst the aminopropylphenyl fragment is found in the heptyl chain one. In 2021, the same team described an analog of **41** as a PqsR antagonist with an IC_50_ of 0.251 µM in a PqsR *E. coli* reporter gene assay. This derivative showed an anti-pyocyanin activity (IC_50_ of 3.65 µM in a *P. aeruginosa* PA14 strain) similar to the one of compound **41**.

The pyranone and pyridinone derivatives **42** and **43** described by Li et al. were both able to inhibit biofilm formation without affecting bacterial growth with IC_50_s of 20.3 and 6.57 µM in a *P. aeruginosa* PAO1 strain, respectively (Scheme 10, Table 4) [117,118]. The compound **42** also revealed an anti-pyocyanin activity with 60% inhibition at 40 µM. It provoked a significant decrease in PqsR autoinduction in a reporter gene assay in *P. aeruginosa* (PAO1-*pqsA-gfp*), but did not affect the production of rhamnolipids and elastases.

The study of PqsR as a potential target for the development of useful AVAs in the therapeutic antibacterial arsenal is still recent (approximately 10 years compared to 20 years for LasR). Actually, only a few HHQ and PQS analogs showed antagonist effect on the PqsR receptor.

##### AQ Autoinducer Non-Analog QSIs

**Scheme 11:**
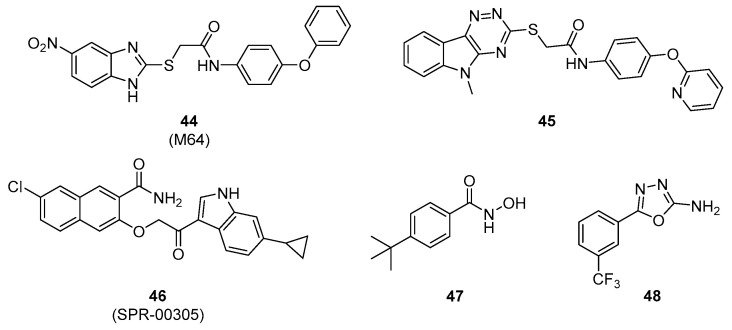
AQ autoinducer non-analog QSIs.

Starkey et al. discovered a promising benzamide–benzimidazole hybrid family of PqsR inhibitors. The lead compound **44** (M64) importantly reduced the production of PQS and HHQ in a *P. aeruginosa* PA14 strain at concentrations in the sub-micromolar range (IC_50_s of 200 nM and 350 nM, respectively) (Scheme 11, Table 5). This compound **44** showed efficient anti-pyocyanin and anti-biofilm properties (IC_50_s of 300 nM and 1 µM, respectively), these are the best anti-virulence activities described in literature. It presented a more potent anti-pyocyanin activity than compound **41** with an IC_50_ of 0.3 vs. 5.9 µM in a photometric quantification PA14 assay. It inhibited the pyocyanin production in various clinical isolates including multi or *pan*drug-resistant strains of *P. aeruginosa*. It is also able to decrease in vitro bacterial tolerance to meropenem and tobramycin by preventing PA14 biofilm formation. This compound **44** binds to PqsR in a PQS non-competitive mode and induces an inactivating conformational modification of the transcription factor. It appears as one of the most promising PqsR inhibitors in preclinical stage. The first in vivo results in PA14-infected mice demonstrated that this new AVA was effective both in monotherapy or in combination with a sub-therapeutic ciprofloxacin treatment to clear acute as well as persistent infections [119].

More recently, Soukarieh et al. described a benzamide–triazinoindole hybrid **45**. It presents a PqsR antagonist activity similar to the benzamide–benzimidazole derivative **44** (IC_50_s of 0.25 and 0.34 µM vs. 0.32 and 1.22 µM, in PAO1-L and PA14 strains, respectively) (Scheme 11, Table 5) [120]. Despite its ability to inhibit pyocyanin production by 80% in PA14 at 1 µM, it failed to restore the ATB activity of ciprofloxacin on mature PAO1-L biofilms, unlike compound **44**.

In 2016, Spero Therapeutics patented an aryloxyacetoindole family of PqsR inhibitors with sub-micromolar activity [121]. Among this family, the indole–naphthalene hybrid **46** (SPR-00305) exhibited the most interesting in vitro anti-QS and anti-pyocyanin efficacy (IC_50_s up to 250 nM in a PA14 strain) (Scheme 11, Table 5). After per os administration, this preclinical QSI induced a substantial decrease in HHQ and PQS production at the infectious site in a PA14-infected mice model (40 and 50% decrease at t = 12 h, respectively) while the compound **44** (M64) was inactive after intravenous administration.

Hartmann’s group also described two families of hydrophilic small-molecule QSIs [122,123]. The benzhydroxamic acid **47** and the 2-amino-oxadiazole **48** appeared as lead compounds with interesting antagonistic PqsR activities in an *E. coli* reporter gene assay (IC_50_s of 12.5 and 7.5 µM, respectively) (Scheme 11, Table 5). The efficiency of these derivatives remained high in a *P. aeruginosa* reporter gene assay (IC_50_s of 23.6 and 38.5 µM, respectively) despite a lower outer membrane permeability of this bacterium. However, these two small aromatic molecules have a worse anti-virulence activity than the biaryl hydrids **44** and **46** (IC_50_ of 87.2 µM for **47** and 46% inhibition at 250 µM for **48**, pyocyanin production in a photometric quantification PA14 assay).

#### 4.3.2. AQ Synthase Inhibitors

The other strategy to block the *pqs* circuit is to inhibit the metabolic biosynthesis pathways of HHQ and PQS by targeting the enzymes involved, such as PqsA, PqsD, and PqsBC.

##### PqsA Inhibitors

**Scheme 12:**
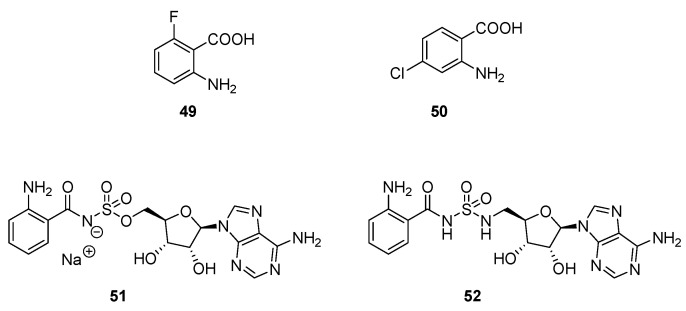
PqsA inhibitors.

The PqsA enzyme plays an important role in the AQ biosynthesis by catalysing the formation of anthraniloyl-CoA from anthranilate (Scheme 4). Coleman et al. demonstrated the interest of the PqsA enzyme as a potential target to quench the *pqs* circuit in *P. aeruginosa* [125]. Indeed, various anthranilate derivatives inhibited the PQS secretion competing with the native substrate for the PqsA active site. Lesic et al. designed PqsA haloanthranilic acid substrate analogs [126]. The introduction of a fluoro or chloro substituent in ortho or para position of the carbonyl group afforded derivatives the ablity to restrict the activation of the carbonyl group for the formation of CoA ester in relation with their donor mesomeric effect. Compounds **49** and **50** drastically reduced the production of HHQ, PQS and pyocyanin in a *P. aeruginosa* PA14 strain at 1.5 mM (Scheme 12, Table 6). Noteworthy, a production of no halogenated AQs was detected, indicating that the metabolic pathway was completely blocked in the presence of these haloanthranilates at sub-inhibitory concentrations. These competitive inhibitors of PqsA improved the survival in a *P. aeruginosa* infected-mice model (10% of survey without treatment vs. 35 and 50% after treatment with **49** and **50**, respectively).

Ji et al. developed new sulfonyladenosine inhibitors by mimicking a PqsA anthranilyl-AMP intermediate substrate (Scheme 12, Table 6) [127]. The anthranilyl-AMS (adenosine monosulfamate) **51** and its sulfamide analog, the anthranilyl-AMSN (adenosine monosulfamate nitrogen) **52** exhibited moderate anti-AQ properties (77 and 92% of PQS secretion inhibition at 1.5 mM in a PA14 strain), but were unable to reduce pyocyanin production. Noteworthy, the outer membrane permeability could be weaker for **51** and **52** than for **49**.

To date, the interest of the two classes of PqsA inhibitors described in the literature as AVAs remains modest.

##### PqsD Inhibitors

**Scheme 13:**
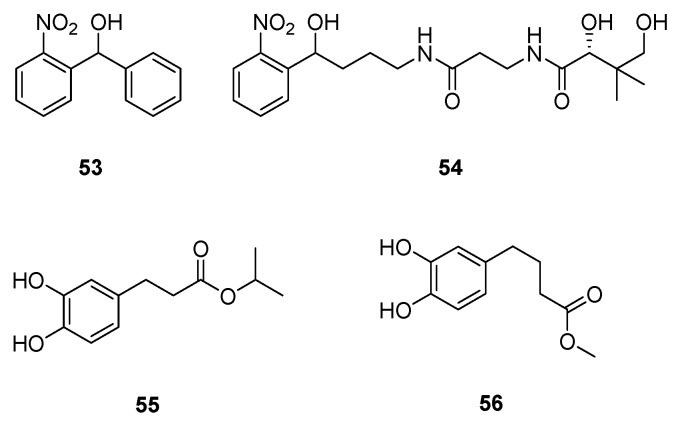
PqsD inhibitors.

The PqsD enzyme involved in the second step of AQ biosynthesis also constitutes an interesting pharmacological target in the *pqs* system (Scheme 4). Following a ligand-based strategy, Hartmann’s team studied several heterocyclic scaffolds as inhibitors. However, although some of them are able to reduce the enzymatic activity of PqsD in vitro, many of these synthesized compounds failed to decrease in cellulo AQ production. This can be explained by a difficulty for these molecules to cross the cell envelop of Gram-negative bacteria due to a low permeability. They can also be *P. aeruginosa* efflux pump substrates or possibly have a lack of PqsD protein binding specificity. Following these results, only two classes of moderately lipophilic small-molecules were optimised as potential AVAs: the (2-nitrophenyl)methanol derivatives **53** and **54** (IC_50_s of 3.2 and 7.9 µM on PqsD, cLogP of 2.47 and 2.15, respectively) and the catechol-based esters **55** and **56** (IC_50_s of 8.6 and 7.9 µM on PqsD, cLogP of 2.77 and 2.47, respectively) (Scheme 13, Table 7) [128,129,130].

The tetrahedral geometry of asymmetric carbon of (2-nitrophenyl)methanol inhibitors **53** and **54** allows their irreversible binding to the PqsD active site, leading to a frozen transition state. Comparatively, the non-competitive anthraniloyl-CoA catechol-based esters **55** and **56** act as shutters of the PqsD active site entrance channel. These QSIs (**53**–**56**) decreased the HHQ biosynthesis in a *P. aeruginosa* PA14 *PqsH^-^* mutant strain at 250 µM, without affecting bacterial growth, by 43, 74, 16 and 18%, respectively. Furthermore, compound **53** reduced the volume of a pre-formed biofilm by 38% after treatment at a concentration of 500 µM during 24 h. Unfortunately, such PqsD inhibitors are susceptible to induce an overproduction of DHQ, a toxic metabolite for eukaryotic cells that contributes to *P. aeruginosa* pathogenicity. Despite interesting in vitro enzymatic PqsD inhibition with IC_50_s in a micromolar range, their anti-QS and anti-virulence properties against the bacteria were revealed as modest.

### 4.4. Multi-Target QSIs

The development of dual-acting QSI potentially capable of blocking the *pqs* circuit at both the gene regulatory and AI metabolic pathways is an effective strategy.

**Scheme 14:**
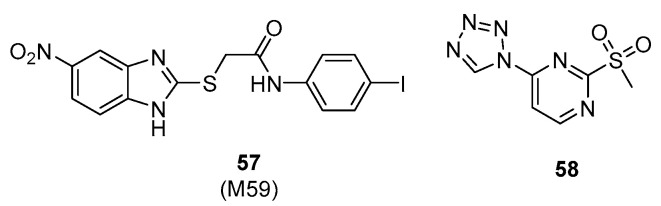
Multi-target QSIs.

PqsBC is the last enzyme involved in the HHQ biosynthesis. It condenses the 2-ABA and octanoyl-CoA into HHQ (Scheme 4). The PqsBC inhibition induces a reduction of HHQ and PQS production that can be accompanied by an increase in 2-AA or DHQ production [131]. However, DHQ and 2-AA are toxic compounds for eukaryotic cells, so the validity of PqsBC as a target for the development of AVAs remains uncertain. Hartmann’s group carried out extended pharmacomodulations on benzamide-benzimidazole PqsR inhibitors that afforded the interesting new derivative **57** (M59). It showed a similar anti-PqsR potency to **44** (M64), but also an additional anti-PqsBC efficiency (IC_50_ of 185 vs. 13.4 µM against PqsBC for M64 vs. M59 following an in vitro biochemical assay, respectively) (Scheme 14, Table 8) [132]. The multi-target compound **57** drastically reduced the production of HHQ and PQS without increasing the formation of cytotoxic DHQ and pro-persistent 2-AA sub-products in contrast to more selective PqsBC inhibitors. Its efficacy to improve survival of human lung epithelial PA14 infected-cells was demonstrated related to its anti-virulence activity. The original profile of this dual PqsR/BC inhibitor makes **57** a promising molecule for the treatment of acute and chronic *P. aeruginosa* infections. Furthermore, by its duality of action, it reduces the risks of bacterial resistance compared to a molecule having only one target.

Another multi-target inhibitor, the tetrazolyl-pyrimidine **58**, revealed dual anti-PqsR/D properties (IC_50_s of 15 and 21 µM on PqsR and PqsD, respectively). This compound possesses synergistic anti-virulence activities on a PA14 strain with anti-pyocyanin and anti-biofilm properties (IC_50_s of 86 and 100 µM, respectively) and pyoverdine secretion inhibition (40% at 100 µM) (Scheme 14, Table 8) [133,134]. The compound **58** also restored the activity of ciprofloxacin under biofilm conditions and it demonstrated an in vivo protective effect in a PA14 infection arthropod model.

The specific profile of *pqs* communication system of *P. aeruginosa* and its primary impact on the bacterial pathogenic phenotype makes PqsR a relevant target for the development of AVAs. To date, several potent PqsR inhibitors such as **44** (M64) and **46** (SPR-00305) enrich this new promising antibacterial arsenal. These two compounds are currently in the preclinical phase. However, the design of specific AQ synthase inhibitors has proven to be more difficult. Nevertheless, the polypharmacology concept is a promising way to avoid the emergence of bacterial resistances.

## 5. Conclusions

The emergence and the dissemination of multidrug-resistant bacteria, especially *P. aeruginosa,* constitute a major public health issue. The QS of this pathogen regulates the secretion of virulence factors and the biofilm formation that are essential for microbial adaptation to environmental changes. Therefore, one of the most interesting approaches to quench *P. aeruginosa* pathogenicity is to target its communication systems. Despite the fact that this is a recent strategy, QS inhibition studies showed promising results for the development of synthetic AVAs and allowed the identification of novel pharmacological targets. The *las* circuit was fully investigated for the development of *pan*-QSIs, but most of the described compounds failed during the preclinic development phase. Furthermore, given the widespread occurrence of AHL-dependent communication systems in the bacterial kingdom, the inhibition of *las* and *rhl* systems has the disadvantage of potentially affecting the host microbiota. On the contrary, the *pqs* circuit based on 2-alkyl-4(1*H*)-quinolone signalling molecules offers a pool of pharmacological tools that specifically target *P. aeruginosa*. Several potent PqsR inhibitors such as **44** (M64) and **46** (SPR-00305) have already been evidenced. These two compounds are currently in preclinical phase. In contrast, the results of specific AQ synthase inhibitors have far been more disappointing. Finally, the development of such innovative QS-quenching anti-virulence agents is a new anti-biotherapy therapeutical strategy of interest.

## Figures and Tables

**Table 1:** Anti-QS and anti-virulence properties of AHL autoinducer analog QSIs.

Class	QSIs	Anti-QS Properties		Anti-Virulence Properties				Ref.
		Target	% Inhibition (IC_50_)	Anti-Biofilm Activity	Anti-Elastase Activity	Anti-Rhamnolipid Activity	Anti-Pyocyanin Activity	
				% Inhibition	% Inhibition	% Inhibition	% Inhibition	
**Ia**	**1**	LasR	80 µM ^(a)^	NA at 250 µM ^(f)^	77% at 250 µM ^(m)^	87% at 250 µM ^(m)^	64% at 250 µM ^(m)^	[81]
		RhlR	90 µM ^(a)^					
	**2**	LasR	≈70% at 100 µM ^(b)^	ND	NA	ND	50% at 25–100µM ^(k)^	[83]
		RhlR	NA at 50 µM ^(c)^					
	**3**	LasR	35% at 100 µM ^(b)^	≈100% at 50 µM ^(g)^	60% at 50 μM ^(j)^	ND	≈100% at 100 µM ^(k)^	
		RhlR	60% at 50 µM ^(c)^					
**Ib**	**4**	LasR	60% at 10 µM and 90% at 100 μM ^(b)^	Weak inhibition at 50 µM ^(g)^	50% at 10 µM ^(j)^	ND	NA at 100 µM ^(k)^	[82]
		RhlR	80% at 50 μM ^(c)^					
	**5**	ND	ND	ND	63% at 200 µM ^(m)^	ND	93% at 200 µM ^(m)^	[85]
	**6**	ND	ND	ND	ND	ND	67% at 200 µM ^(m)^	
	**7**	ND	ND	ND	34% at 200 µM ^(m)^	ND	73% at 200 µM ^(m)^	
**IIa**	**8**	LasR	154 μM ^(d)^	ND	ND	ND	34% à 100 µM ^(l)^	[86]
**IIb**	**9**	LasR	12.5 µM ^(b)^	Strong inhibition at 50 µM ^(h)^	66% at 200 μM ^(m)^	ND	ND	[80,87,88]
	**10**	LasR	≈25 µM ^(b)^	Strong inhibition at 50 µM ^(h)^	77% at 200 µM ^(m)^	ND	≈57% à 50 µM ^(k)^	
	**11**	LasR	ND	36.2% at 15 µM ^(i)^	13.7% at 15 µM ^(m)^	28.1% at 15 µM ^(m)^	34.5% at 15 µM ^(m)^	[89]
**IIIa**	**12**	LasR	0.79 µM ^(e)^	ND	ND	ND	ND	[90]
	**13**	LasR	0.14 µM ^(e)^	ND	ND	ND	ND	
**IIIb**	**14**	LasR	0.40 µM ^(e)^	ND	ND	ND	ND	
**IV**	**15**	ND	ND	40% at 162.5 µM ^(i)^	16% at 162.5 µM ^(m)^	57% at 162.5 µM ^(m)^	22% at 162.5 µM ^(m)^	[91]

NA = Not active; ND = Not determined; Anti-QS evaluations carried out using reporter gene assays on/with (a) *P. aeruginosa* (PAO1); (b) *P. aeruginosa* (PAO-JP2)/competition against odDHL (1 µM); (c) *P. aeruginosa* (PAO-JP2)/competition against a combination of odDHL (1µM) and BHL (10 µM); (d) *P. aeruginosa* (PAO-JP2); (e) *E. coli* (DH5α, pJN105L + pSC11)/competition with odDHL (1 μM); Anti-virulence evaluations carried out on/by (f) *P. aeruginosa* (PAO1)/confocal laser scanning microscopy; (g) static biofilm assay with *P. aeruginosa* (PAO-JP2) in the presence of a combination of odDHL (1 µM) and BHL (10 µM)/confocal laser scanning microscopy; (h) static biofilm assay with *P. aeruginosa* (PAO1(pLVAgfp))/composite 3D micrography; (i) static biofilm assay with *P. aeruginosa* (PAO1)/crystal violet dyeing (CV) and optical density (OD) measurement; (j) static biofilm assay with *P. aeruginosa* (PAO-JP2) in the presence of a combination of odDHL (5 µM) and BHL (10 µM)/OD measurement; (k) *P. aeruginosa* (PAO-JP2) in the presence of a combination of odDHL (25 µM) and BHL (25 µM) or without AI/OD measurement; (l) *P. aeruginosa* (PA14)/OD measurement and (m) *P. aeruginosa* (PAO1)/OD measurement.

**Table 2:** Anti-QS and anti-virulence properties of AHL autoinducer non-analog QSIs.

QSIs	Anti-QS Properties		Anti-Virulence Properties			Ref.
	Target	% Inhibition (IC_50_)	Anti-Biofilm Activity	Anti-Elastase Activity	Anti-Pyocyanin Activity	
			% Inhibition	% Inhibition	% Inhibition	
**16** (C56)	LasR	>50% at 11 µM ^(a)^	60% at 0.5 µM ^(g)^	Active at 0.5 µM ^(j)^	ND	[92,93]
**17** (C30)	ND	ND	ND	ND	ND	[93]
**18**	LasR	Active above 40 µM ^(b)^	74% at 256 µg/mL ^(h)^	ND	10% at 64 µg/mL ^(k)^	[94]
**19**	LasR	10 µM ^(c)^	ND	60% at 100 µM ^(l)^	90% at 100 µM ^(l)^	[95]
**20**	LasR	30 nM ^(c)^	ND	20% at 10 µM ^(l)^	40% at 10 µM ^(l)^	
**21**	LasR	50 µM ^(d)^	ND	ND	ND	[96]
**22**	LasR	70% at 4 mg/mL ^(e)^	76% at 4 mg/mL ^(i)^	75% (LasA) and 65% (LasB) at 4 mg/mL ^(l)^	68% at 4 mg/mL ^(l)^	[97]
**23**	LasR	64% at 62.5 µM ^(f)^	ND	ND	ND	[98]

ND = Not Determined; Anti-QS evaluations carried out using reporter gene assays on/with (a) *P. aeruginosa* (PAO-JP2, *lasB-gfp(ASV)*)/competition against odDHL (100 nM); (b) *P. aeruginosa* (PAO1, *lasB-gfp*); (c) *P. aeruginosa* (MW1, pUM15, LasR-dependent promoter *prsaL*)/competition against odDHL (0.3 µM); (d) *P. aeruginosa* (MW1, pUM15); (e) *E. coli* (MG4, pKDT1719); (f) *P. aeruginosa* (MH602); **Anti-virulence evaluations** carried out on/by (g) static biofilm assay with *P. aeruginosa* (PAO1)/confocal laser scanning microscopy; (h) static biofilm assay with *P. aeruginosa* (PAOA, ATCC27853)/CV dyeing and OD measurement; (i) static biofilm assay with *P. aeruginosa* (PAO1)/OD measurement and confocal laser scanning microscopy; (j) *P. aeruginosa* (PAO-JP2) in the presence of odDHL (1 µM) and BHL (3 µM)/OD measurement; (k) *P. aeruginosa* (PAOA)/OD measurement; (l) *P. aeruginosa* (PAO1)/OD measurement.

**Table 3:** Anti-QS and anti-virulence properties of RhlR-modulating QSIs.

QSIs	Anti-QS Properties		Anti-Virulence Properties			Ref.
	Target	% Inhibition/Activation (IC_50_/EC_50_)	Anti-Biofilm Activity	Anti-Rhamnolipid Activity	Anti-Pyocyanin Activity	
			% Inhibition	% Inhibition	IC_50_	
**24**	RhlR (agonist)	43% at 1 mM ^(a)^	≈40% at 100 µM ^(e)^	ND	8 µM ^(g)^	[100]
		80% at 20 µM ^(b)^				
**25**	RhlR (agonist)	4.5 µM ^(c)^	ND	ND	ND	[101]
**26**	RhlR (agonist)	7.2 µM ^(c)^				
**27**	RhlR (antagonist)	20% at 10 µM ^(d)^	15% at 10 µM ^(f)^	≈40% at 10 µM ^(h)^	NA ^(h)^	[105]

NA = Not active, ND = Not Determined; Anti-QS evaluations carried out using reporter gene assays on/with (a) *E. coli* (*rhlA-gfp*)/competition against odDHL (100 nM); (b) *E. coli* (*rhlA–gfp*); (c) *P. aeruginosa* (PAO-JP2, *prhlILVAGFP*); (d) *P. aeruginosa* (PAO1, *rhlA-lacZ*); Anti-virulence evaluations carried out on/by (e) static biofilm assay with *P. aeruginosa* (PA14)/confocal laser scanning microscopy; (f) static biofilm assay with *P. aeruginosa* (PAO1)/CV dyeing and OD measurement; (g) *P. aeruginosa* (PA14)/OD measurement; (h) *P. aeruginosa* (PAO1)/OD measurement.

**Table 4:** Anti-QS and anti-virulence properties of AQ autoinducer analog QSIs.

QSIs	Anti-QS Properties	Anti-Virulence Properties				Ref.
	IC_50_	Anti-Biofilm Activity	Anti-Elastase Activity	Anti-Rhamnolipid Activity	Anti-Pyocyanin Activity	
		% Inhibition (IC_50_)	% Inhibition (IC_50_)	% Inhibition (IC_50_)	% Inhibition (IC_50_)	
**28**	51 nM ^(a)^	ND	ND	ND	ND	[106]
**29**	54 nM ^(a)^	ND	NA at 5 µM ^(m)^	NA at 5 µM ^(m)^	74% at 3 mM ^(m)^	
**30**	51 nM ^(a)^	ND	ND	ND	1.9 µM ^(m)^	[107]
	1.322 µM ^(b)^					
**31**	5 µM ^(c)^	ND	ND	ND	50 µM ^(n)^	[109]
**32**	313 nM ^(d)^	ND	ND	ND	23% à 939 nM ^(o)^	[110]
	342 nM ^(e)^					
**33**	289 nM ^(d)^	ND	ND	ND	36% at 867 nM ^(o)^	
	265 nM ^(e)^					
**34**	ND	40% at 140 µM ^(h)^	ND	ND	140 µM ^(m)^	[112]
**35**	ND	50% at 125 µM ^(h)^	ND	ND	2.5 µM ^(m)^	
**36**	25 µM ^(f)^	50 µM ^(i)^	35% at 50 µM ^(m or n)^	ND	12 µM ^(m)^	[113]
**37**	30 µM ^(f)^	20% at 100 µM ^(i)^	30% at 50 µM ^(m or n)^	ND	25 µM ^(m)^	
**38**	2.3 µM ^(e)^	8 µM ^(j)^	ND	ND	65% at 8 µM ^(m)^	[114]
	12.4 µM ^(d)^	34 µM ^(k)^			80% at 34 µM ^(o)^	
**39**	≥50 nM ^(a)^	≥500 nM ^(l)^	ND	ND	≥250 nM ^(m)^	[115]
**40**	≥50 nM ^(a)^	≥500 nM ^(l)^	ND	ND	Between 251 and 1000 nM ^(m)^	
**41**	0.14 µM ^(a)^	ND	ND	ND	5.9 µM ^(m)^	[116]
**42**	Active from 2.5 to 40 µM ^(g)^	20 µM ^(i)^	NA ^(n)^	NA ^(n)^	60% at 40 µM ^(n)^	[117]
**43**	Active from 1.25 to 20 µM ^(g)^	6.57 μM ^(i)^	Active from 1.25 to 20 µM ^(n)^	Active from 1.25 to 20 µM ^(n)^	Active from 1.25 to 20 µM ^(m)^	[118]

NA = Not Active; ND = Not Determined; Anti-QS evaluations carried out using reporter gene assays on/with (a) *E. coli* (DH5, pEAL08-2)/competition with PQS (50 nM); (b) *P. aeruginosa* (PA14, pEAL08-2)/competition with PQS (50 nM); (c) *P. aeruginosa* (PAO1, miniCTX::pqsA’-lux fusion); (d) *P. aeruginosa* (PAO1-L, CTX:PpqsA-lux); (e) *P. aeruginosa* (PA14, CTX:PpqsA-lux); (f) *P. aeruginosa* (PAO1 ΔpqsA, CTX lux::pqsA)/competition with HHQ (5 µM); (g) *P. aeruginosa* (PAO1, *pqsA-gfp*); Anti-virulence evaluations carried out on/by (h) static biofilm assay with *P. aeruginosa* (PAO1)/electronic microscopy; (i) static biofilm assay with *P. aeruginosa* (PAO1)/CV dyeing and OD measurement; (j) static biofilm assay with *P. aeruginosa* (PA14, mCTX::PpqsA-lux)/confocal laser scanning microscopy; (k) static biofilm assay with *P. aeruginosa* (PAO1-L, mCTX::PpqsA-lux)/confocal laser scanning microscopy; (l) static biofilm assay with *P. aeruginosa* (PA14)/CV dyeing and OD measurement; (m) *P. aeruginosa* (PA14)/OD measurement; (n) *P. aeruginosa* (PAO1)/OD measurement; (o) *P. aeruginosa* (PAO1-L)/OD measurement.

**Table 5:** Anti-QS and anti-virulence properties of AQ autoinducer non-analog QSIs.

QSIs	Anti-QS Properties		Anti-Virulence Properties		Ref.
	Target	IC_50_	Anti-Biofilm Activity	Anti-Pyocyanin Activity	
			IC_50_	% Inhibition (IC_50_)	
**44** (M64)	PqsR	0.32 µM ^(a)^	1 µM ^(c)^	300 nM ^(d)^	[119,120,124]
		1.22 µM ^(b)^			
**45**	PqsR	0.25 µM ^(a)^	ND	80% at 1 µM ^(e)^	[120]
		0.34 µM ^(b)^			
**46**	ND	ND	ND	0.05–0.25 µΜ ^(d)^	[121]
**47**	PqsR	12.5 µM ^(f)^ 23.6 µM ^(g)^	ND	87.2 µM ^(d)^	[122,123]
**48**	PqsR	7.5 µM ^(f)^ 38.5 µM ^(g)^	ND	46% at 250 μM ^(d)^	[122]

ND = Not Determined; Anti-QS evaluations carried out using reporter gene assays on (a) *P. aeruginosa* (PAO1-L, mCTX:P*pqsA-lux*); (b) *P. aeruginosa* (PA14, mCTX:P*pqsA-lux*); Anti-virulence evaluations carried out on/by (c) static biofilm assay with *P. aeruginosa* (PA14)/CV dyeing and OD measurement; (d) *P. aeruginosa* (PA14)/OD measurement; (e) *P. aeruginosa* (PA14, mCTX:P*pqsA-lux*)/OD measurement; (f) *E. coli* (DH5α pEAL08-2) in presence of PQS (50nM); (g) *P. aeruginosa* (PA14 ΔpqsA, pEAL08-2) in presence of PQS (50 nM).

**Table 6:** Anti-QS and anti-virulence properties of PqsA inhibitors.

QSIs	Anti-QS Properties		Anti-Virulence Properties	Ref.
	Anti-HHQ Activity	Anti-PQS Activity	Anti-Pyocyanin Activity	
	% Inhibition	% Inhibition	% Inhibition	
**49**	≈100% at 1.5 mM ^(a)^	≈100% at 1.5 mM ^(a)^	≈100% at 1.5 mM ^(b)^	[126]
**50**	≈100% at 1.5 mM ^(a)^	≈100% at 1.5 mM ^(a)^	≈100% at 1.5 mM ^(b)^	
**51**	67% at 1.5 mM ^(a)^	77% at 1.5 mM ^(a)^	NA at 1 mM ^(b)^	[127]
**52**	90% at 1.5 mM ^(a)^	92% at 1.5 mM ^(a)^	NA at 1 mM ^(b)^	

ND = Not Determined; Anti-QS evaluations carried out on/by (a) *P. aeruginosa* (PA14)/LC-MS monitoring; Anti-virulence evaluations carried out on/by (b) *P. aeruginosa* (PA14)/OD measurement.

**Table 7:** Anti-QS and anti-virulence properties of PqsD inhibitors.

QSIs	Anti-QS Properties	Anti-Virulence Properties	Ref.
	IC_50_	Anti-Biofilm Activity	
		% Inhibition	
**53**	3.2 µM ^(a)^	38% at 500 µM ^(b)^	[128,129]
**54**	7.9 µM ^(a)^	ND	
**55**	8.6 µM ^(a)^	ND	[130]
**56**	7.9 µM ^(a)^	ND	

ND = Not Determined; Anti-QS evaluations carried out using reporter gene assays on (a) *E. coli* (pT28b(þ)/*pqsD*) in presence of anthranilyl-CoA (5 μM) and β-ketodecanoic acid (70 μM); Anti-virulence evaluations carried out on/by (b) static biofilm assay with *P. aeruginosa* (PA14)/3D micrography.

**Table 8:** Anti-QS and anti-virulence of multi-target QSIs.

QSIs	Anti-QS Properties		Anti-Virulence Properties		Ref.
	Target	IC_50_	Anti-Biofilm Activity	Anti-Pyocyanin Activity	
			IC_50_	IC_50_	
**57** (M59)	PqsBC	13.4 µM ^(a)^	ND	ND	[132]
	PqsR	ND			
**58**	PqsR	15 µM ^(b)^	100 µM ^(d)^	86 µM ^(c)^	[133,134]
	PqsD	21 µM ^(c)^			

ND = Not Determined; Anti-QS evaluations carried out using reporter gene assays on/with (a) PqsBC enzyme in presence of 2-ABA and octanoyl-CoA; (b) *E. coli* (DH5α, pEAL08-2); (c) PqsD enzyme in presence of anthranilyl-CoA (5 μM) and β-ketodecanoic acid (70 μM); Anti-virulence evaluations carried out on/by (d) static biofilm assay with *P. aeruginosa* (PA14)/CV dyeing and OD measurement; (e) *P. aeruginosa* (PA14)/OD measurement.

## Data Availability

Data sharing not applicable.

## Abbreviations

ABC	ATP binding cassette
ACP	Acyl carrier protein
AIs	Autoinducers
AIPs	Autoinducing peptides
AHL	*N*-acyl-homoserine lactone
AQs	2-alkyl-4(1*H*)-quinolones
AQNOs	2-alkyl-4-quinolone *N*-oxides
ATB	Antibiotic
AVAs	Anti-virulence agents
BHL	*N*-butanoyl-L-homoserine lactone
CA	Chorismic acid
CIO	Cyanide-insensitive terminal oxidase
DHQ	2,4-dihydroxyquinoline
ECDC	European Centre for Disease Prevention and Control
eDNA	Extracellular DNA
EPS	Exopolysaccharides
ETA	Exotoxin A
EU/EEA	European Union/European Economic Area
HCL	HomoCysteine Lactone
HHQ	2-heptyl-4(1*H*)-quinolone
HQNO	2-heptyl-4-quinolone *N*-oxide
HSL	HomoSerine Lactone
IgA	Immunoglobulin A
IgG	Immunoglobulin G
LPS	Lipopolysaccharide
MDR	Multidrug-resistant
MvfR	Multiple virulence-factor regulator
NQNO	2-nonyl-4-quinolone *N*-oxide
odDHL	*N-*(3-oxododecanoyl)-L-homoserine lactone
OMV	Outer membrane vesicles
PCA	Phenazine-1-carboxylic acid
Pe	Phosphatidylethanolamine
PG	Phosphatidylglycerol
PIV	Type IV proteases
PLLs	Phosphotriesterase-like lactonases
PQS	*Pseudomonas* quinolone signal
QQ	Quorum Quenching
QS	Quorum Sensing
QSIs	Quorum Sensing inhibitors
SOD	Superoxide dismutase
sPLA2-IIA	Soluble Type IIA phospholipase A2
